# Derivation of asthma severity from electronic prescription records using British thoracic society treatment steps

**DOI:** 10.1186/s12890-022-02189-3

**Published:** 2022-11-03

**Authors:** Holly Tibble, Aziz Sheikh, Athanasios Tsanas

**Affiliations:** 1 Usher Institute, Edinburgh Medical School, University of Edinburgh, Edinburgh, UK; 2 Asthma UK Centre for Applied Research, Edinburgh, UK; 3 Health Data Research UK BREATHE Hub for Respiratory Health, University of Edinburgh, Edinburgh, UK; 4 The Alan Turing Institute, London, UK; 5 Usher Institute, NINE Bioquarter, University of Edinburgh, 9 Little France road, EH16 4UX Edinburgh, France

**Keywords:** Asthma, Severity, Electronic Health Records, Pharmacotherapy, Pharmacoepidemiology, Treatment guidelines

## Abstract

**Background:**

Asthma severity is typically assessed through a retrospective assessment of the treatment required to control symptoms and to prevent exacerbations. The joint British Thoracic Society and Scottish Intercollegiate Guidelines Network (BTS/SIGN) guidelines encourage a stepwise approach to pharmacotherapy, and as such, current treatment step can be considered as a severity categorisation proxy. Briefly, the steps for adults can be summarised as: no controller therapy (Step 0), low-strength Inhaled Corticosteroids (ICS; Step 1), ICS plus Long-Acting Beta-2 Agonist (LABA; Step 2), medium-dose ICS + LABA (Step 3), and finally either an increase in strength or additional therapies (Step 4). This study aimed to investigate how BTS/SIGN Steps can be estimated from across a large cohort using electronic prescription records, and to describe the incidence of each BTS/SIGN Step in a general population.

**Methods:**

There were 41,433,707 prescriptions, for 671,304 individuals, in the Asthma Learning Health System Scottish cohort, between 1/2009 and 3/2017. Days on which an individual had a prescription for at least one asthma controller (preventer) medication were labelled *prescription events.* A rule-based algorithm was developed for extracting the strength and volume of medication instructed to be taken daily from free-text data fields. Asthma treatment regimens were categorised by the combination of medications prescribed in the 120 days preceding any prescription event and categorised into BTS/SIGN treatment steps.

**Results:**

Almost 4.5 million ALHS prescriptions were for asthma controllers. 26% of prescription events had no inhaled corticosteroid prescriptions in the preceding 120 days (Step 0), 16% were assigned to BTS/SIGN Step 1, 7% to Step 2, 21% to Step 3, and 30% to Step 4. The median days spent on a treatment step before a step-down in treatment was 297 days, whereas a step-up only took a median of 134 days.

**Conclusion:**

We developed a reproducible methodology enabling researchers to estimate BTS/SIGN asthma treatment steps in population health studies, providing valuable insights into population and patient-specific trajectories, towards improving the management of asthma.

**Supplementary information:**

The online version contains supplementary material available at 10.1186/s12890-022-02189-3.

## Background

An individual’s asthma severity can be considered as underlying and static feature, independent of current symptoms, typically measured by asthma control in the absence of therapy [1, 2]. As such, it may confound analyses of statistical associations if not appropriately estimated and controlled for.

Asthma severity has been defined previously in different contexts to include lung function and the risk of acute exacerbations [3–5], but it may also be indicated by the minimum treatment required to achieve control at a specific time [1, 6–9]. This approach has been recommended by the joint American Thoracic Society/European Respiratory Society Taskforce [10].

Asthma treatment progression is typically considered to be a linear process, progressively recommending more advanced treatments (known as a *step up*) if adequate control is not reached at a previous step [11, 12]. Asthma severity can thus be objectively ranked using stepwise treatment guidelines, such as those developed jointly by the British Thoracic Society (BTS) and the Scottish Intercollegiate Guidelines Network (SIGN) [11] and those detailed in the Global INitiative for Asthma (GINA) report [12]. Both guidelines encourage an incremental approach to pharmacotherapy beginning with low-strength Inhaled CorticoSteroid (ICS) monotherapy, and progressively increasing medication strength and/or adding new therapies if asthma control is not achieved. The BTS/SIGN recommendations [11] for the progression of treatment are described in Appendix A.

Categorisation of asthma severity may be used in clinical practice guidance [13–16], and clinical trials [17, 18], as well as in research. In epidemiological studies, the categorisation of asthma severity is often used as a confounder for the effect of some exposure to the risk of clinical outcomes such as asthma attacks [19–25], or as the outcome itself when investigating the role of exposures in asthma development [5, 26, 27].

Previous studies using the BTS/SIGN steps [8, 22, 28, 29] have often faced some combination of three key weaknesses. First, not all possible or observed regimens map to an explicit treatment step, and thus require ad-hoc judgements to be made. Secondly, their methods were often not sufficiently transparent for validation or to be reproduced by other researchers. Finally, they often required custom data collection, such as for patients to report their current treatment regimen.

Electronic Health Records (EHRs) can be used in pragmatic observational and intervention studies of asthma across wide populations [6], without the need for intervention or time-consuming data collection, and with better reproducibility. It also enables algorithms to be incorporated into a *learning healthcare system*, in which patient data are used to generate a continuous loop of knowledge-generation, evidence based clinical practice change, and change assessment/validation [30, 31]. However, estimating BTS/SIGN steps from EHRs is a challenging multi-faceted task: it requires identifying asthma prescribed medications, conducting free-text analysis on General Practitioners’ (GPs) clinical records, and operationalising the BTS/SIGN steps from extracted data.

The aim of this study was to develop and present a reproducible methodology for classifying asthma severity by proxy of BTS/SIGN treatment steps using prescription EHRs, to further population-level asthma research, and to describe their incidence in the general population. The classification of patients in a cohort into severity groups permits adjustment for confounding in formal statistical tests, which grants a more accurate and precise estimate of factors which influence asthma outcomes, and thus better knowledge to inform clinical asthma care and management.

## Methods

### Overview of study design

In this study, we conducted a secondary cohort analysis of a Scottish prescribing EHR dataset, as described in the following section. We identified prescribing records pertaining to asthma controller medications, and extracted pertinent information from the structured and free-text data fields, such as medication type, brand, and dose. Treatment regimens (the combination of prescribed therapies) were estimated at each day on which an individual had a prescription for at least one asthma medication (a *prescription day)* from the prescriptions issued to that individual in the previous 120 days. 120 days was chosen to balance sufficient time for a refill of each regimen component to have been filled, facilitating accurate treatment regimen identification, but short enough that seasonal changes in regimen (or strength of ICS, for example) could be detected. Each regimen was the assigned to its corresponding treatment step in the adult BTS/SIGN Guidelines [11].

### Data

The Asthma Learning Healthcare System (ALHS) dataset [30] recruited over half a million patients from 75 general practices in Scotland, with primary care records linked to national accident and emergency, hospital and mortality datasets using the Scottish health identification number known as the Community Health Index (CHI) [32]. As such, we have approximately 10% population coverage [30]. No demographic exclusion criteria were implemented, and so this cohort can be considered approximately representative of the Scottish general practice-registered population: that is, approximately 70% of the population live in urban areas, 14% in rural areas, 8% are aged 75 or o over, 15% are aged 14 and under, and 50% are female [33, 34].

The fields available in the prescription dataset were: pseudo-anonymised patient study identifier (such as “ID0001” – allowing linkage between datasets and for observations within datasets, without revealing the identity of the individual), date of prescription, date of dispensing, medication name, BNF item code, formulation, prescribed quantity, dispensed quantity, and free-text native dose instructions.

### Identifying asthma medications

To identify asthma medications, we matched the medication name recorded in the prescription record to a lookup table listed in Appendix B containing the medications and brand names. We have only included asthma medications which are licensed in the UK. Corticosteroids can also be used in other dosages and formulations for conditions such as allergic rhinitis [35] and Crohn’s disease [36]. Therefore, ICS medications with spray or drop formulation were excluded, along with records containing keywords listed in Appendix C in the dose directions or medication name.

The brand names of the medications were also checked to exclude brands of inhaled medications for related conditions such as Chronic Obstructive Pulmonary Disease (COPD), or for nasal sprays with missing formulation (Appendix C).

The designated category of medication was updated, such that corticosteroid solutions were distinguished from inhaled formulations by listed formulation “SOL” or by the presence of any of the following keywords in the dose directions or medication name: “SACHET”, “RESPULE”, “NEB”, “VIAL”, or “AMPOULE”.

### Asthma medication data cleaning

The BTS/SIGN treatment steps are a categorisation based on the type and dosage of medications a person has been prescribed. To estimate the prescribed daily dose, we needed to calculate the number of daily doses (*dose frequency*), the number of puffs per dose (*dose quantity*), and the strength of each puff, recorded in the prescription record.

#### Dose frequency

When the dose frequency for a particular medication is clearly indicated based on keywords and phrases (listed in Appendix F, e.g., terms such as ‘once’ or ‘4 times’), these values can easily be extracted. However, inferring non-explicitly defined prescribing patterns EHRs is less straightforward. There is research literature suggesting that the dose frequency is primarily dictated by the pharmacokinetic profile of medications [37]. For example, some medications have longer half-life which would indicate that they would not be regularly prescribed to be taken multiple times a day. Therefore, we have decided to impute dose frequencies using the most common (mode) dose frequency for a particular medication type (such as ‘budesonide’) as a reasonable approach to impute missing entries.

#### Dose quantity

Secondly, the dose quantity was estimated by searching for the numbers one, two, three, or four (in numerals and written out) preceded by “take” or “inhale”, or followed by “daily”, “at”, “to be taken”, or “puf” (with a single ‘f’ to allow for typographical errors), or “p” (followed by a space; ‘p’ being commonly used as shorthand for puffs). When this information could not be extracted, the mode by medication type was imputed.

#### Medication strength

Medication strength was extracted by searching the free-text prescription information for any of the following dosages in micrograms [10 000, 5000, 4000, 2000, 1000, 500, 400, 320, 250, 200, 184, 160, 125, 100, 92, 80, 65, 50], followed by “MCG” or “MICROGRAM”, with and without spaces between the value and phrase. Additionally, for ICS + LABA medications, which have strengths for the ICS and LABA components separately, the values could proceed “/” (without a space). By searching through the values in descending order we ensured that “250 MCG” had not been extracted as “50 MCG”, for example. Following that, we searched for the following values in milligrams [0.5, 20, 10, 5, 4, 2, 1] followed by “MG” or “MILLIGRAM” (again, with or without a space between). Similarly, 0.5 was searched prior to the integer values such that “0.5MG” is not extracted as “5MG”. Missing strengths were imputed using the mode by medication type. The extracted strength in micrograms for a prescription was compared to the lookup table presented in Appendix B to flag values outside of the range of strengths prescribed for specifically for asthma, indicating that it should be excluded.

### British thoracic Society Treatment Step classification

#### Strength classification

The 2019 Adult BTS/SIGN Guidelines[11] present a single value for each level of dosage: low, medium, or high. In practice, many regimens did not perfectly align with these guidelines. As such, conversion of the continuous ICS and ICS/LABA daily dose into the three levels (low, medium, and high) was based on ranges, accommodating all observed values as listed in Appendix E. An additional category is also presented for paediatric treatment (“*very low dose”*), which is typically around half of the ‘low dose’ value, and would thus fall into the ‘low dose’ range. Medications no longer recommended for prescription (and thus not included in the BTS/SIGN guidelines, such as AeroBec, Beclazone, and Filair) were grouped with other medications of the same drug (such as beclometasone) and inhaler (dry powder inhaler or metered dose inhaler) type.

Medium-dose was assigned from one microgram higher than the low-dose value up to the medium-dose value unless there was no recommended low-dose. In this case, half of the medium-dose value was used as the lower range limit. Similarly, the high-dose category was assigned from one microgram higher than the medium-dose value up to four times the medium-dose value, unless the medium-dose value was missing, in which case half of the high-dose value was used as the lower range limit and twice the high-dose value for the upper range limit. If the medication strength value recorded was above the upper limit of the high-dose level, then the medication strength category ‘unknown’ was assigned.

#### Asthma regimen classification

Treatment step was calculated on any day on which an individual had a prescription for at least one asthma medication (a *prescription day)*, based on any medications which had been prescribed in the last 120 days. A run-in period of 120 days (January 31st to June 1st, 2009) allowed refills of different medications to be accumulated for the first regimen estimate. However, it is a natural limitation that we are not able to distinguish between a complex regimen, and a sudden change in regimen resulting in new treatments having been added while existing treatments have not been finished.

#### Regimen to treatment step mapping

**Fig. 1:**
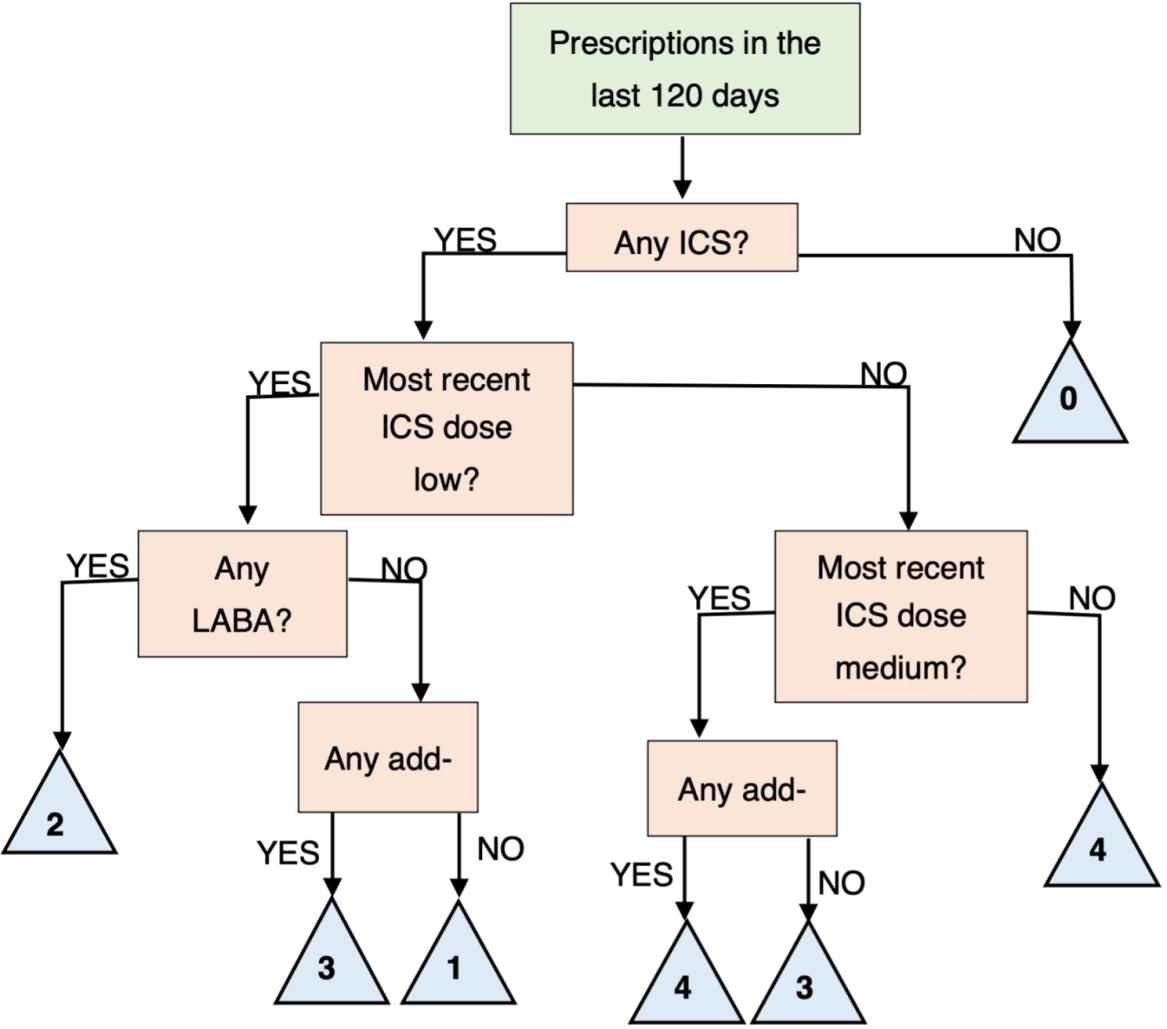
Decision tree demonstrating the implementation of the Adult British Thoracic Society and Scottish Intercollegiate Guidelines Network treatment steps

Notes: ICS = Inhaled CorticoSteroid, LABA = Long-Acting Beta-2 Agonist.

There is only one possible regimen at Step 1 (low-dose ICS) for adults, but higher numbers of variants at the higher steps. The 2019 BTS/SIGN guidelines recommend a minimum treatment of as-needed low dose ICS (Step 1), and thus we have categorised regimens without any ICS component as treatment Step 0, as shown in Fig. 1. These include SABA monotherapy, which is only recommended “for those with infrequent short-lived wheeze” [11]. The guidelines highlight that there is some evidence to support the use of ICS alternatives, such as LTRA or theophylline as the primary controller, although they are not listed in the explicit treatment step disambiguation presented in their Fig. 2 [11].

Although not presented here, the steps for children are typically the same as the adult steps, but one strength category down. For example, Step 1 for children is very low dose ICS, rather than low dose ICS as for adults. For those aged under 5 years, LTRA may be used in place of ICS for children aged under 5, and LABA is not recommended.

### Analysis plan

Having assigned the treatment steps, we measured the time spent on a treatment step before changing, stratified by whether the change was a step up or down in intensity. An individual’s final treatment step change was omitted from these calculations as the time to change was censored (the duration before change after the end of the study was not known). The rate of change and the frequency of changes in regimen, moving with and between treatment steps, was also assessed.

The Strengthening the Reporting of Observational Studies in Epidemiology (STROBE) guidelines [38] were used to guide the presentation of the methodology and results used herein, and to ensure that no important information had been omitted. R scripts for data processing and analysis are available at https://github.com/hollytibble/BTS_Step_Derivation.

## Results

### Asthma medication data cleaning

As described in the prescription processing report presented in Appendix F, 4,450,709 of the 41,433,707 prescriptions in the dataset (10.7%) were identified as eligible asthma medications, after having removed prescriptions with dates outside of the extracted study period (n = 673), prescriptions which noted they should be deleted (n = 39), prescriptions which did not match any asthma brand names or ingredients (n = 36,264,262), prescriptions which were excluded based on formulation (n = 716,472), and prescriptions which matched non-asthma indication brand names (n = 1332).

Multiple prescriptions on one day were condensed such that there was only one BTS/SIGN step or regimen per person on a single day, resulting in 2,880,546 records. Additionally, records before June 1st, 2009, were excluded to allow a run-in period of regimen estimation from staggered refills, resulting in 2,772,818 records for 157,503 unique individuals. There were 625,998 person-years containing at least one prescription.

**Fig. 2:**
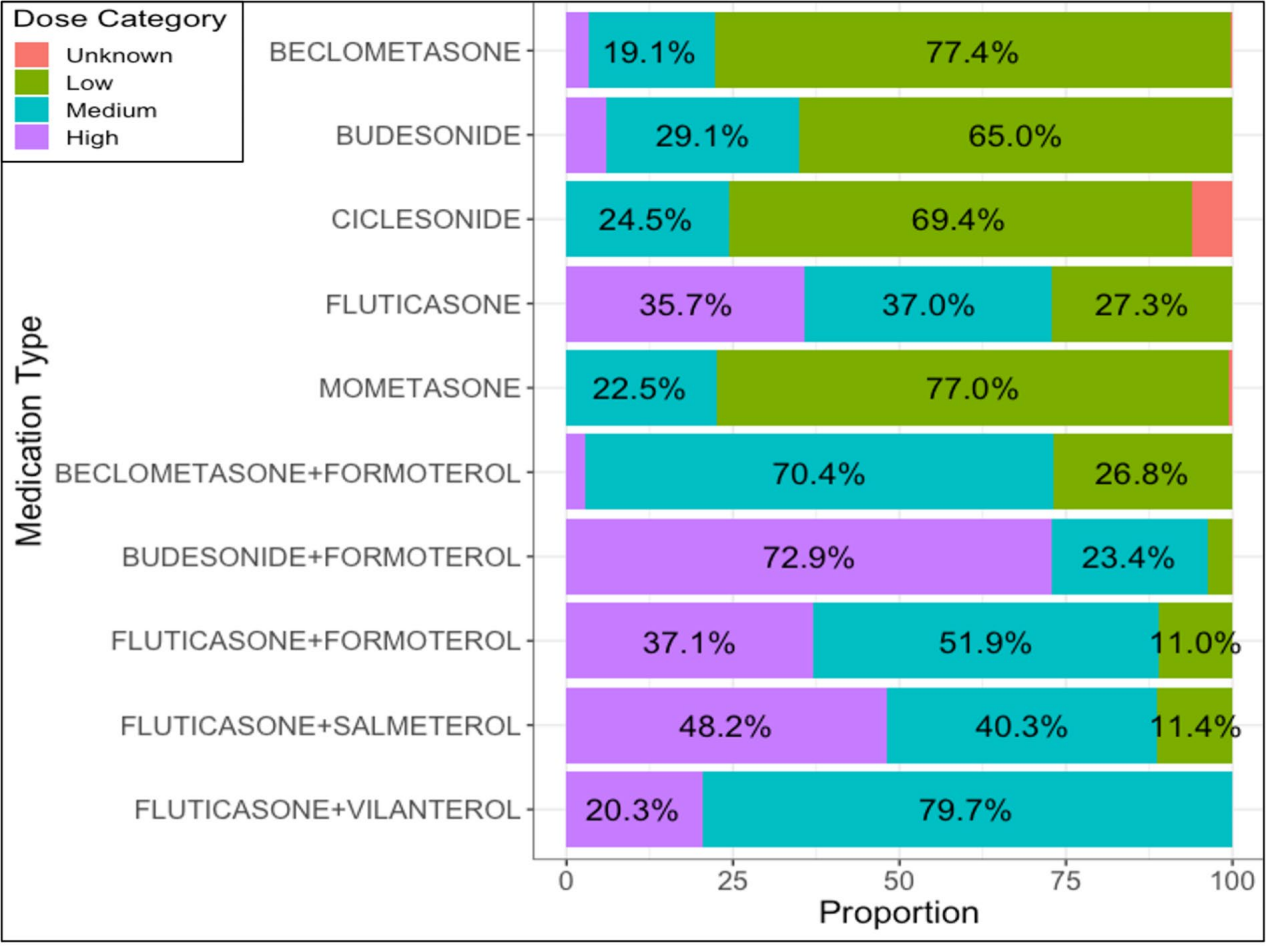
Percentage of Inhaled Corticosteroid and combination Long-Acting Beta-2 Agonist prescriptions assigned to each dose category by medication type

ICS medications of the medium and high dose categories were more commonly prescribed as combination ICS + LABA inhalers (Fig. 2). Ciclesonide had the highest proportion of prescriptions outside of the recommended range (6.1%).

### British thoracic society treatment step classification

The first step to assigning the treatment steps to a patient’s treatment was to identify the regimen, the combination of treatments prescribed concurrently, that each individual was on at the time of any new prescription being written (either repeat or first instance). There were 110 unique regimens observed, categorised by ICS strength category, and other therapies in the preceding 120 days. 19.0% of prescription events (n = 526,239) corresponded to the regimen high-dose ICS + LABA (either standalone or combination inhalers). Table 1 shows the 10 regimens which corresponded to more than 1% of the total prescription events, with the other 100 regimens comprising 232,286 prescription events (8.4%).

Next, these regimens were mapped to the treatment steps, using the decision tree in Fig. 1. Only one regimen was assigned to treatment Step 1, 13 to Step 2, 11 to Step 3, and 53 to Step 4. The remaining 32 regimens were assigned to Step 0. These include both SABA monotherapy (17.8% of prescription events), which is only recommended “for those with infrequent short-lived wheeze” [11], and LABA monotherapy (5.8% of prescription events), which is contraindicated in the BTS/SIGN guidelines currently. The guidelines highlight that there is some evidence to support the use of ICS alternatives, such as LTRA or theophylline as the primary controller, although they are not listed in the explicit treatment step disambiguation.

**Table 1:** Asthma Treatment Regimens by Prescription Events

Regimen	Number of Prescription Events (Percentage)
High-strength ICS + LABA	526,239 (19.0%)
SABA	494,009 (17.8%)
Low-strength ICS	438,955 (15.8%)
Medium-strength ICS + LABA	427,567 (15.4%)
Low-strength ICS + LABA	176,630 (6.4%)
LABA	160,630 (5.8%)
Medium-strength ICS	127,642 (4.6%)
High-strength ICS + LABA + LTRA	88,746 (3.2%)
Medium-strength ICS + LABA + LTRA	58,119 (2.1%)
High-strength ICS + LABA + Theophylline	41,784 (1.5%)

Notes: ICS = Inhaled Corticosteroid, LABA = Long-Acting Beta-2 Agonist, SABA = Short-Acting Beta-2 Agonist, LTRA = Leukotriene Receptor Antagonist

Next, we examined the regimens which were assigned to step 0, by virtue of not aligning with the treatment guidelines and having no ICS component. There were 2,772,818 prescription events, of which 715,016 were for non-ICS prescriptions, with no ICS prescriptions in the previous 120 days, and were thus assigned Step 0. For prescription events assigned to step 0, 69.1% had been prescribed only SABA for the last 120 days, 22.5% had been prescribed only LABA, and 3.7% had been prescribed only LTRA. The remaining 4.7% of step 0 prescription events corresponded to regimens which had prevalence lower than 2%

25.8% of prescription events (n = 715,016) were assigned to Step 0, 15.8% of prescription events (n = 438,955) were Step 1, 7.1% (n = 198,163) were Step 2, 21.0% (n = 583,483) were Step 3, and 30.2% (n = 837,201) were Step 4.

### Characteristics of treatment step changes

16.4% of changes in regimen were within the same treatment step as the previous regimen, 39.9% were a step down in treatment, and 43.7% were a step up. Changes within a treatment step were more common on steps 0 (15.6% of regimen changes) and 4 (39.4%) compared to steps 2 (7.9%) and 3 (9.0%). Step 1 comprised only one regimen and so it was not possible to change regimen within the same step.

**Fig. 3:**
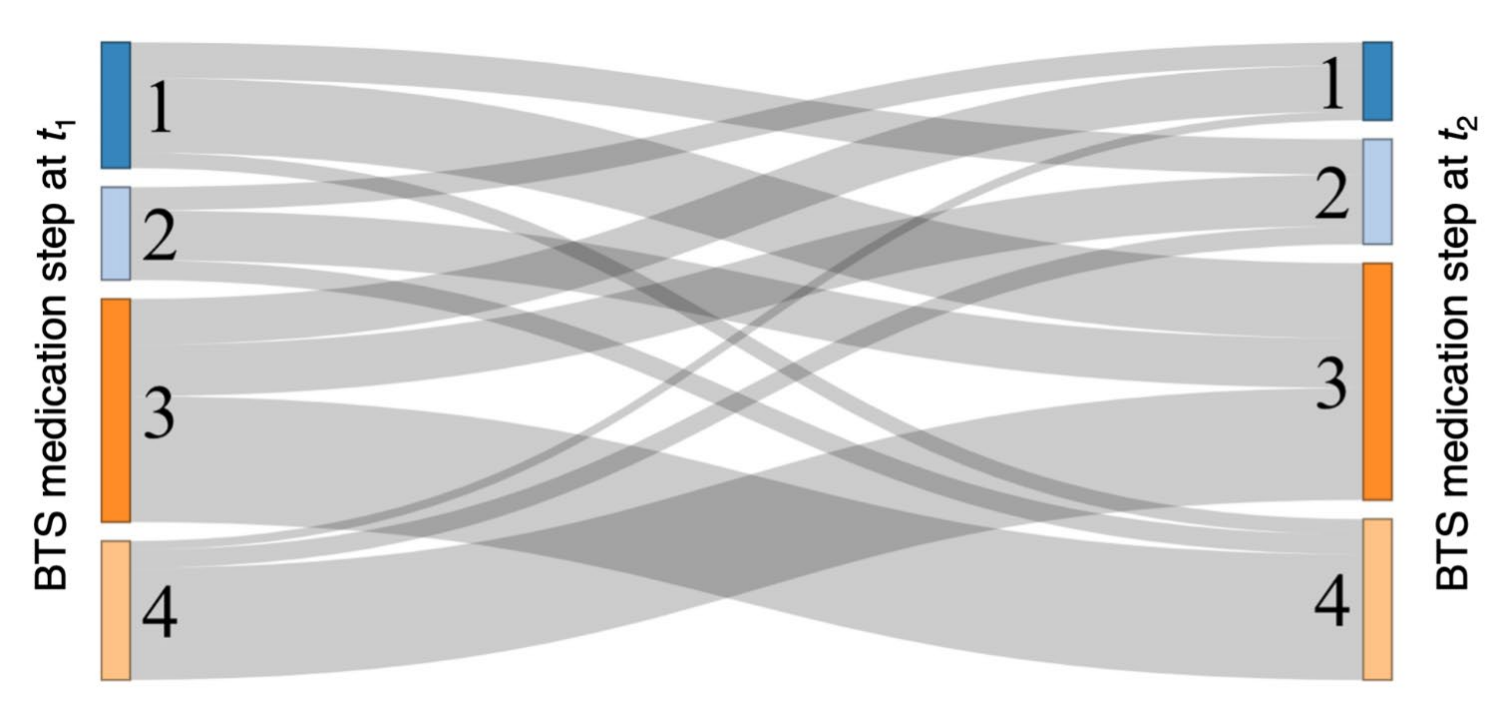
Sankey Plot of British Thoracic Society and Scottish Intercollegiate Guidelines Network treatment Step Changes (Excluding changes to and from Step 0)

Notes: *t*_1_ and *t*_2_ indicate the time before and after a prescribing event, respectively. Transitions to and from step 0 are omitted as they often reflected periods of non-adherence rather than clinician-sanctioned changes in regimen.

Most changes in BTS/SIGN step were by a single step (65.7% of step ups and 71.9% of step downs) and most changes were between steps 3 and 4, with only 10.1% were between steps 1 and 2 (Fig. 3).

There were 625,998 person-years containing at least one prescription, of which for 79.1%, individuals stayed on the same treatment step throughout. 18.7% of person-years featured two distinct treatment steps, of which 24.8% featured multiple switches between the two steps. Only 2.2% of person-years featured three or more distinct treatment steps. 7.2% of person-years featured three or more changes.

Overall, the median time between starting and changing treatment step was 206 days (interquartile range 83 to 514 days). The time duration on a treatment step was typically longer if the change was a step down (median 297 days, interquartile range 152–647 days) compared to a step up (134 days, interquartile range 48–393 days).

The median time to change was substantially shorter for Step 0 than other steps (Table 2) although this step had the highest percentage of censored records (the duration before change after the end of the study was not known; 47.1%) which may have lowered the median estimate.

**Table 2:** Duration (days) until British Thoracic Society and Scottish Intercollegiate Guidelines Network Treatment Step Change

BTS/SIGN Step	Duration until Step Change (days)		Percent Censored
	**Median**	**Interquartile Range**	
0	127	46–365	47.1
1	312	150–660	35.2
2	223	121–492	23.7
3	204	85–505	26.9
4	273	136–648	29.1

## Discussion

### Principal findings

We have developed a reproducible methodology towards categorising asthma treatment regimens and classifying asthma severity by proxy of BTS/SIGN treatment steps using prescription EHRs. This classification process enables population-level studies to examine and adjust for the role of severity in association and outcome studies, and to improve the quality of research which drives clinical asthma management.

We assigned regimens based on combinations of prescribed medications that overlapped in 120-day periods, and found that almost one in five prescription events (19%) corresponded to the regimen high-strength ICS plus LABA, either in a single combination inhaler or in two distinct inhaler units.

There were 625,998 person-years containing at least one prescription. 30% of prescription events corresponded to a regimen at BTS/SIGN step 4, 21% at step 3, 6% at step 2, 6% at step 1, and 26% to step 0 (no ICS prescriptions in the previous 120 days). People stayed on one treatment step for a median of 200 days, but the duration was typically longer if the change was a step down (median 294 days) compared to a step up (129 days). Only 2.7% of person-years featured more than two distinct treatment steps.

### Results in context

Asthma treatment classifications such as the BTS/SIGN steps and the GINA steps have been used previously for both population-level [8, 22] and individual-level analyses [32, 39, 40]. Previous studies using the BTS/SIGN steps have interpreted and implemented the guidelines in different ways [8, 22, 28, 29], and indeed the guidelines have also been updated over time, making direct comparisons challenging. There are four main strengths of the methods described herein for the identification of asthma treatment regimens, and their mapping to BTS/SIGN treatment steps.

First, our implementation of the treatment guidelines maps all possible regimens to a treatment step, which removes the requirement for any manual assignment as there are no possible unclassified combinations. Charlton et al. [28], for example, noted that not all observed regimens “*directly translate to a specific treatment step*”, and so their allocation was based on “*the most comparable treatment step”*, although this process was not formally defined. Our implementation is intuitive to interpret, as demonstrated by the decision tree presented in Fig. 1.

Secondly, the use of a grace period (the look-back window for prior prescriptions to classify the treatment regimen) allows prescriptions for different components to be collected on different dates without the treatment step being incorrectly estimated. The use of a 120-day period enables detection of regimen changes in finer resolution, which can be used to evaluate rates of clinical outcomes by treatment step, unlike the year-long observation period used in such studies as Bloom et al. [8]. This may also facilitate the detection of seasonal changes in prescribing patterns. The grace period used in this study is sufficiently long, however, to capture some degree of *as-needed* ICS use, which is encouraged at step 1 of the 2019 guidelines [11]. Most ICS are prescribed in 30-day supplies (120-dose cannisters for two puffs twice daily, or 60-dose cannisters for two puffs once daily) [41–43], and thus up to 25% usage would still be captured as continuation.

BTS/SIGN treatment steps are not explicitly recorded in EHRs, hence the necessity for this methodology, however this also limits our ability to validate our classification against any gold standard. We were able, however, to compare our values extracted from the free-text fields of the prescription records to those from the methodology of McTaggart et al. [44]; an algorithm for the extraction of prescription data from the free-text prescribing fields which was applied automatically to all research datasets extracted from the Scottish Prescribing Information System (PIS). A substantial limitation of their approach is that it does not adapt well to the nuances of asthma pharmacotherapy, such as combined therapy inhalers, and has not been made available for researchers to adapt. When both methods had managed to obtain values for the number of doses per day, the amount to take at each dose, and the strength of the medication, the agreement was between 99.6 and 99.9%. Our methods consistently resulted in lower missingness (before imputation): 13% versus 10% for daily dose frequency, 13% versus 11% for dose quantity, and 62% versus 8% for dose strength. The most common phrases which were not translated (no information extracted) were “Morning and night” (equalling two daily dose times), “[*n*] inhalations” or “[*n]* inspirations” (equalling *n* units of inhaled medication to be taken at each dose time), and ICS + LABA medications such as Seretide and Symbicort which were commonly listed without the unit (i.e., “SERETIDE 250”).

Finally, an important strength of this analysis is the transparency of our methods. The mapping from prescription records to time-varying BTS step estimate described herein was derived using Scottish electronic health records, which contain rich free-text dose directions, often not available in other UK and international research datasets. The text-processing components of the methods are not always possible to perfectly implement in other settings, therefore, however by providing detailed descriptions of the rule-based imputation process it is possible to implement robustly in lieu of this data. We hope that the availability of the derivation code for use by other researchers (link provided in the methods section) will facilitate better future population-level studies, to improve asthma patient care and management.

### Limitations and future work

The methods described herein are exclusively designed for the classification of large EHR study populations into treatment-based categories, as a proxy for asthma severity. The treatment classification process should not be used to guide patient care.

The primary aim of this study was to develop a reproducible methodology for classifying asthma severity by proxy of BTS/SIGN treatment steps using prescription EHRs, We have focussed herein on the Adult BTS/SIGN recommendations, however the steps can easily be expanded to cover the Paediatric recommendations. No linkage to primary care records has been conducted, and thus this population will include children who will have been classified at a lower treatment step than if the population was stratified.

To ascertain a patient’s current treatment regimen most accurately, we would be required to regularly ask them which medications they are currently taking. In lieu of this, we have devised a methodology making use of EHRs, which can be applied easily to large-scale populations with limited expense or time for data collection. This is naturally an imperfect process, however. One key limitation is that regimens which overlap within the 120-day grace period (changes commenced before a previous regimen had expired) would be considered as components of a single, complex, regimen. Tracking sudden changes to regimen or treatment step over time within a patient can be utilised for error detection, however.

In this study, we observed that clinicians were faster to step-up the prescribed treatment (a median of just over 4 months) than to step-down (a median of around 9.5 months). This might have been because asthma medication reviews were often prompted by patient reported symptoms [45], or because GPs were keen to quickly achieve symptom control (and, conversely, reticent to lose it). GPs may also have concerns about adherence to current therapy [46]. Identifying the patient-specific factors associated with determinants of poor outcomes resulting from treatment step-downs may provide insights into personalised risk-benefit assessment at medication reviews. It is important that this work acknowledges the crucial role that patient adherence to therapy plays on asthma control.

The data linkage between prescribing and dispensing records in Scottish EHRs (conducted by National Services Scotland Information Services Division) is an imperfect process, as prescriptions containing multiple items have only a single identifier, rather than an item-specific identifier. As such, if the items are listed in a different order on the dispensing and prescribing records, additional information relating to a specific item (such as dosing direction notes from the pharmacist) may be assigned to the wrong prescription item. Although rare, this mismatch likely led to a small number asthma-related records being erroneously excluded on the basis of indication, as they contained exclusion keywords, or having an incorrect value assigned for the strength, daily dose frequency, or dose quantity.

In the Methods (‘Identifying Asthma Medications’), we described how efforts were made to exclude medications used for alternative indications, including Crohn’s Disease and COPD, as well as asthma. However, the high incidence (5.8% of all prescription events) of LABA only prescriptions (which are not recommended for asthma, but may be recommended for COPD) indicated in Table 1 highlight the distinct probability that generic therapies included herein may have not been intended for asthma management. However, it may also simply reflect cases in which the BTS/SIGN guidelines are not being applied with regards to pharmacological management. Prior to applying the proposed methodology towards asthma severity classification, a diagnosis of asthma should be required to restrict the analysis population.

As discussed in the Background, the primary motivation for this work to facilitate adjustment for potential confounding from asthma severity in inferential analyses mapping patient characteristics to clinical outcome risk [32]. It may also be utilised as an outcome for studies which wish to identify exposures which may contribute to the development of more severe asthma [5, 26, 27]. Finally, the treatment steps and the statistics related to change between steps can be used to identify population-level differences in clinical care.

Data extraction from the free-text fields of the drug description and instructions followed easy to implement approaches: the guiding principle was to develop something which should be straightforward to operationalise. Future work could integrate more advanced Natural Language Processing (NLP) techniques to investigate this further.

## Conclusion

The novel and reproducible methodology presented herein (and the accompanying R scripts) enable researchers to easily replicate BTS/SIGN asthma treatment steps. These steps can be used to efficiently estimate the severity of asthma in population-level studies, and to demonstrate changes of symptom severity over time using routinely collected prescription EHRs.

## Electronic supplementary material

Below is the link to the electronic supplementary material.

Supplementary Material 1

Supplementary Material 2

## Data Availability

The ALHS data are held by the National Services Scotland electronic Data Research and Innovation Service (eDRIS) in the National Safe Haven (phs.edris@phs.scot). To be able to access the ALHS data, researchers must be added to the study team, and have their analysis plan approved by the PBPP. They must also have passed the Safe Users of Research data Environment (SURE) training, provided by the Administrative Data Research Network (ADRN).

## **Abbreviations**

BTS: British Thoracic Society
SIGN: Scottish Intercollegiate Guidelines Network
GINA: Global INitiative for Asthma
ICS: Inhaled CorticoSteroid
EHRs: Electronic Health Records
GPs: General Practitioners?
ALHS: Asthma Learning Healthcare System
CHI: Community Health Index
LABA: Long-Acting Beta-2 Agonist
COPD: Chronic Obstructive Pulmonary Disease
LTRA: Leukotriene Receptor Antagonist
NLP: Natural Language Processing

## References

[1] Taylor DR, Bateman ED, Boulet LP, Boushey HA, Busse WW, Casale TB (2008). A new perspective on concepts of asthma severity and control. Eur Respir J.

[2] Vollmer WM. Assessment of asthma control and severity. Ann Allergy, Asthma Immunol [Internet]. 2004;93(5):409–14. Available from: 10.1016/S1081-1206(10)61406-8. 10.1016/S1081-1206(10)61406-8 15562878

[3] Bousquet J, Mantzouranis E, Cruz AA, Aït-Khaled N, Baena-Cagnani CE, Bleecker ER (2010). Uniform definition of asthma severity, control, and exacerbations: Document presented for the World Health Organization Consultation on Severe Asthma. J Allergy Clin Immunol.

[4] Mukherjee M, Nwaru BI, Soyiri I, Grant I, Sheikh A. High health gain patients with asthma: a cross-sectional study analysing national Scottish data sets. Prim Care Respir Med [Internet]. 2018 [cited 2018 Sep 5];28:27. Available from: www.nature.com/npjpcrm. 10.1038/s41533-018-0094-6 PMC6053372 30026587

[5] de Marco R, Marcon A, Jarvis D, Accordini S, Almar E, Bugiani M (2006). Prognostic factors of asthma severity: A 9-year international prospective cohort study. J Allergy Clin Immunol.

[6] Varsano S, Segev D, Shitrit D. Severe and non-severe asthma in the community: A large electronic database analysis. Respir Med [Internet]. 2017;123:131–9. Available from: 10.1016/j.rmed.2016.12.017. 10.1016/j.rmed.2016.12.017 28137489

[7] Papaporfyriou A, Papaioannou AI, Hillas G, Konstantelou E, Tseliou E, Koulouris N, et al. Inflammatory profile in optimally treated patients with adult versus early-onset asthma. Postgrad Med [Internet]. 2019;131(5):324–9. Available from: 10.1080/00325481.2019.1600884. 10.1080/00325481.2019.1600884 30920326

[8] Bloom CI, Nissen F, Douglas IJ, Smeeth L, Cullinan P, Quint JK (2018). Exacerbation risk and characterisation of the UK’s asthma population from infants to old age. Thorax.

[9] Hull SA, McKibben S, Homer K, Taylor SJ, Pike K, Griffiths C (2016). Asthma prescribing, ethnicity and risk of hospital admission: An analysis of 35,864 linked primary and secondary care records in East London. npj Prim Care Respir Med.

[10] Reddel HK, Taylor DR, Bateman ED, Boulet LP, Boushey HA, Busse WW (2009). An official American Thoracic Society/European Respiratory Society statement: Asthma control and exacerbations - Standardizing endpoints for clinical asthma trials and clinical practice. Am J Respir Crit Care Med.

[11] British T, Society SIGN. British guideline on the management of asthma (2019 Edition). 2019.

[12] Global Initiative for Asthma. Pocket Guide for Asthma Management and Prevention 2019 [Internet]. 2019 [cited 2019 Dec 9]. Available from: www.ginasthma.org.

[13] Bleecker ER, Menzies-Gow AN, Price DB, Bourdin A, Sweet S, Martin AL (2020). Systematic literature review of systemic corticosteroid use for asthma management. Am J Respir Crit Care Med.

[14] Song WJ, Lee JH, Kang Y, Joung WJ, Chung KF (2019). Future risks in patients with severe asthma. Allergy Asthma Immunol Res.

[15] NICE. Omalizumab for treating severe persistent allergic asthma. 2013.

[16] Mulgirigama A, Barnes N, Fletcher M, Pedersen S, Pizzichini E, Tsiligianni I. A review of the burden and management of mild asthma in adults — Implications for clinical practice. Respir Med [Internet]. 2019;152(April):97–104. Available from: 10.1016/j.rmed.2019.04.024. 10.1016/j.rmed.2019.04.024 31128617

[17] Chupp G, Laviolette M, Cohn L, McEvoy C, Bansal S, Shifren A, et al. Long-term outcomes of bronchial thermoplasty in subjects with severe asthma: a comparison of 3-year follow-up results from two prospective multicentre studies. Eur Respir J [Internet]. 2017;50(2):1700017. Available from: 10.1183/13993003.00017-2017. 10.1183/13993003.00017-2017 PMC5593347 28860266

[18] Murray CS, Foden P, Sumner H, Shepley E, Custovic A, Simpson A (2017). Preventing severe asthma exacerbations in children a randomized trial of mite-impermeable bedcovers. Am J Respir Crit Care Med.

[19] Østrem A, Horne R. Reducing asthma attacks: consider patients’ beliefs. Nat Publ Gr [Internet]. 2015 [cited 2017 Dec 21];25:15021. Available from: https://www.ncbi.nlm.nih.gov/pmc/articles/PMC4532155/pdf/npjpcrm201521.pdf. 10.1038/npjpcrm.2015.21 PMC4532155 25833035

[20] Schatz M, Meckley LM, Kim M, Stockwell BT, Castro M. Asthma Exacerbation Rates in Adults Are Unchanged Over a 5-Year Period Despite High-Intensity Therapy. J Allergy Clin Immunol Pract [Internet]. 2014;2(5):570–4. Available from: 10.1016/j.jaip.2014.05.002. 10.1016/j.jaip.2014.05.002 25213050

[21] Turner SW, Murray C, Thomas M, Burden A, Price DB. Applying UK real-world primary care data to predict asthma attacks in 3776 well-characterised children: a retrospective cohort study. npj Prim Care Respir Med [Internet]. 2018;28:28. Available from: http://www.nature.com/articles/s41533-018-0095-5. 10.1038/s41533-018-0095-5 PMC6056517 30038222

[22] Blakey JD, Price DB, Pizzichini E, Popov TA, Dimitrov BD, Postma DS, et al. Identifying Risk of Future Asthma Attacks Using UK Medical Record Data: A Respiratory Effectiveness Group Initiative. J Allergy Clin Immunol Pract [Internet]. 2017;5(4):1015–24. Available from: 10.1016/j.jaip.2016.11.007. 10.1016/j.jaip.2016.11.007 28017629

[23] Price D, Wilson A, Chisholm A, Rigazio A, Burden A, Thomas M, et al. Predicting frequent asthma exacerbations using blood eosinophil count and other patient data routinely available in clinical practice. J Asthma Allergy [Internet]. 2016;9:1. Available from: https://www.dovepress.com/predicting-frequent-asthma-exacerbations-using-blood-eosinophil-count--peer-reviewed-article-JAA. 10.2147/JAA.S97973 PMC4708874 26793004

[24] Tanaka A, Uno T, Sato H, Jinno M, Hirai K, Miyata Y, et al. Predicting future risk of exacerbations in Japanese patients with adult asthma: A prospective 1-year follow up study. Allergol Int [Internet]. 2017;66(4):568–73. Available from: 10.1016/j.alit.2017.02.013. 10.1016/j.alit.2017.02.013 28318883

[25] Nwaru BI, Shah SA, Tibble H, Pillinger R, McLean S, Ryan D, et al. Hormone Replacement Therapy and Risk of Severe Asthma Exacerbation in Perimenopausal and Postmenopausal Women: 17-Year National Cohort Study. J Allergy Clin Immunol Pract [Internet]. 2021; Available from: 10.1016/j.jaip.2021.02.052. 10.1016/j.jaip.2021.02.052 33705997

[26] Comhair SAA, Gaston BM, Ricci KS, Hammel J, Dweik RA, Teague WG (2011). Detrimental effects of environmental tobacco smoke in relation to asthma severity. PLoS ONE.

[27] Rage E, Siroux V, Künzli N, Pin I, Kauffmann F (2009). Air pollution and asthma severity in adults. Occup Environ Med.

[28] Charlton RA, Hutchison A, Davis KJ, de Vries CS. Asthma Management in Pregnancy. PLoS One. 2013;8(4). 10.1371/journal.pone.0060247 PMC3617219 23593182

[29] Gayle A, Tebboth A, Pang M, Guelfucci F, Argoubi R, Sherman S, et al. Real-life prescribing of asthmatic treatments in UK general practice over time using 2014 BTS/SIGN steps. npj Prim Care Respir Med [Internet]. 2019;29(1):1–7. Available from: 10.1038/s41533-019-0137-7. 10.1038/s41533-019-0137-7 PMC6624291 31296867

[30] Soyiri IN, Sheikh A, Reis S, Kavanagh K, Vieno M, Clemens T, et al. Improving predictive asthma algorithms with modelled environment data for Scotland: an observational cohort study protocol. BMJ Open [Internet]. 2018 [cited 2018 Sep 30];8:e23289. Available from: http://bmjopen.bmj.com/. 10.1136/bmjopen-2018-023289 PMC5961591 29780034

[31] Friedman CP, Rubin J, Brown J, Buntin M, Corn M, Etheredge L, et al. Toward a science of learning systems: a research agenda for the high-functioning Learning Health System. J Am Med Informatics Assoc [Internet]. 2015 Jan 1 [cited 2022 May 31];22(1):43–50. Available from: https://academic.oup.com/jamia/article/22/1/43/834511. 10.1136/amiajnl-2014-002977 PMC4433378 25342177

[32] Tibble H, Tsanas A, Horne E, Horne R, Mizani M, Simpson CR, et al. Predicting asthma attacks in primary care: protocol for developing a machine learning-based prediction model. BMJ Open [Internet]. 2019 Jul 1 [cited 2019 Aug 13];9(7):e028375. Available from: https://bmjopen.bmj.com/content/9/7/e028375. 10.1136/bmjopen-2018-028375 PMC6624024 31292179

[33] National Records of Scotland. Mid-Year Population Estimates Scotland, Mid-2016 Population estimates by sex, age and area. 2017.

[34] Mulholland RH, Vasileiou E, Simpson CR, Robertson C, Ritchie LD, Agrawal U, et al. Cohort Profile: Early Pandemic Evaluation and Enhanced Surveillance of COVID-19 (EAVE II) Database Who is in the cohort? IEA Int Epidemiol Assoc Int J Epidemiol [Internet]. 2021;1064–5. Available from: https://academic.oup.com/ije/article/50/4/1064/6294008. 10.1093/ije/dyab028 PMC8195245 34089614

[35] Scadding GK, Kariyawasam | HH, Scadding | G, Mirakian | R, Buckley | RJ, Rotiroti | G (2017). BSACI guideline for the diagnosis and management of allergic and non-allergic rhinitis (Revised Edition 2017; First edition 2007). Clin Exp Allergy.

[36] Martins R, Carmona C, George B, Epstein J. Management of Crohn’s disease: summary of updated NICE guidance. BMJ [Internet]. 2019 Nov 1 [cited 2021 Sep 20];367:l5940. Available from: https://www.bmj.com/content/367/bmj.l5940. 10.1136/bmj.l5940 31676715

[37] Derendorf H, Nave R, Drollmann A, Cerasoli F, Wurst W (2006). Relevance of pharmacokinetics and pharmacodynamics of inhaled corticosteroids to asthma. Eur Respir J.

[38] Benchimol EI, Smeeth L, Guttmann A, Harron K, Moher D, Petersen I, et al. The REporting of studies Conducted using Observational Routinely-collected health Data (RECORD) Statement. PLOS Med [Internet]. 2015 Oct 6 [cited 2019 Sep 30];12(10):e1001885. Available from: 10.1371/journal.pmed.1001885. 10.1371/journal.pmed.1001885 PMC4595218 26440803

[39] Bateman ED, Buhl R, O’Byrne PM, Humbert M, Reddel HK, Sears MR (2015). Development and validation of a novel risk score for asthma exacerbations: The risk score for exacerbations. J Allergy Clin Immunol.

[40] Hussain Z, Shah SA, Mukherjee M, Sheikh A. Predicting the risk of asthma attacks in children, adolescents and adults: protocol for a machine learning algorithm derived from a primary care-based retrospective cohort. BMJ Open [Internet]. 2020 [cited 2021 Feb 4];10:e036099. Available from: http://bmjopen.bmj.com/. 10.1136/bmjopen-2019-036099 PMC7380838 32709646

[41] Suruki RY, Daugherty JB, Boudiaf N, Albers FC (2017). The frequency of asthma exacerbations and healthcare utilization in patients with asthma from the UK and USA. BMC Pulm Med.

[42] Laforest L, Belhassen M, Devouassoux G, Didier A, Ginoux M, Van Ganse E. Long-Term Inhaled Corticosteroid Adherence in Asthma Patients with Short-Term Adherence. J Allergy Clin Immunol Pract [Internet]. 2016;4(5):890–899.e2. Available from: 10.1016/j.jaip.2016.07.008. 10.1016/j.jaip.2016.07.008 27587320

[43] Blais L, Vilain A, Kettani F-Z, Forget A, Lalonde G, Beauchesne M-F, et al. Accuracy of the days’ supply and the number of refills allowed recorded in Québec prescription claims databases for inhaled corticosteroids. BMJ Open [Internet]. 2014;4:5903. Available from: http://bmjopen.bmj.com/. 10.1136/bmjopen-2014-005903 PMC4248090 25432902

[44] McTaggart S, Nangle C, Caldwell J, Alvarez-Madrazo S, Colhoun H, Bennie M (2018). Use of text-mining methods to improve efficiency in the calculation of drug exposure to support pharmacoepidemiology studies. Int J Epidemiol.

[45] Price C, Agarwal G, Chan D, Goel S, Kaplan AG, Boulet LP (2019). Large care gaps in primary care management of asthma: A longitudinal practice audit. BMJ Open.

[46] Chipps BE, Bacharier LB, Murphy KR, Lang D, Farrar JR, Rank M, et al. The Asthma Controller Step-down Yardstick. Ann Allergy, Asthma Immunol [Internet]. 2019;122(3):241–262.e4. Available from: 10.1016/j.anai.2018.12.004. 10.1016/j.anai.2018.12.004 30550809

