# Supplementary figures and images for: Derivation of asthma severity from electronic prescription records using British thoracic society treatment steps
Source: BMC Pulm Med. 2022 Nov 3;22:397. doi: 10.1186/s12890-022-02189-3 (PMC9635147; doi:10.1186/s12890-022-02189-3)

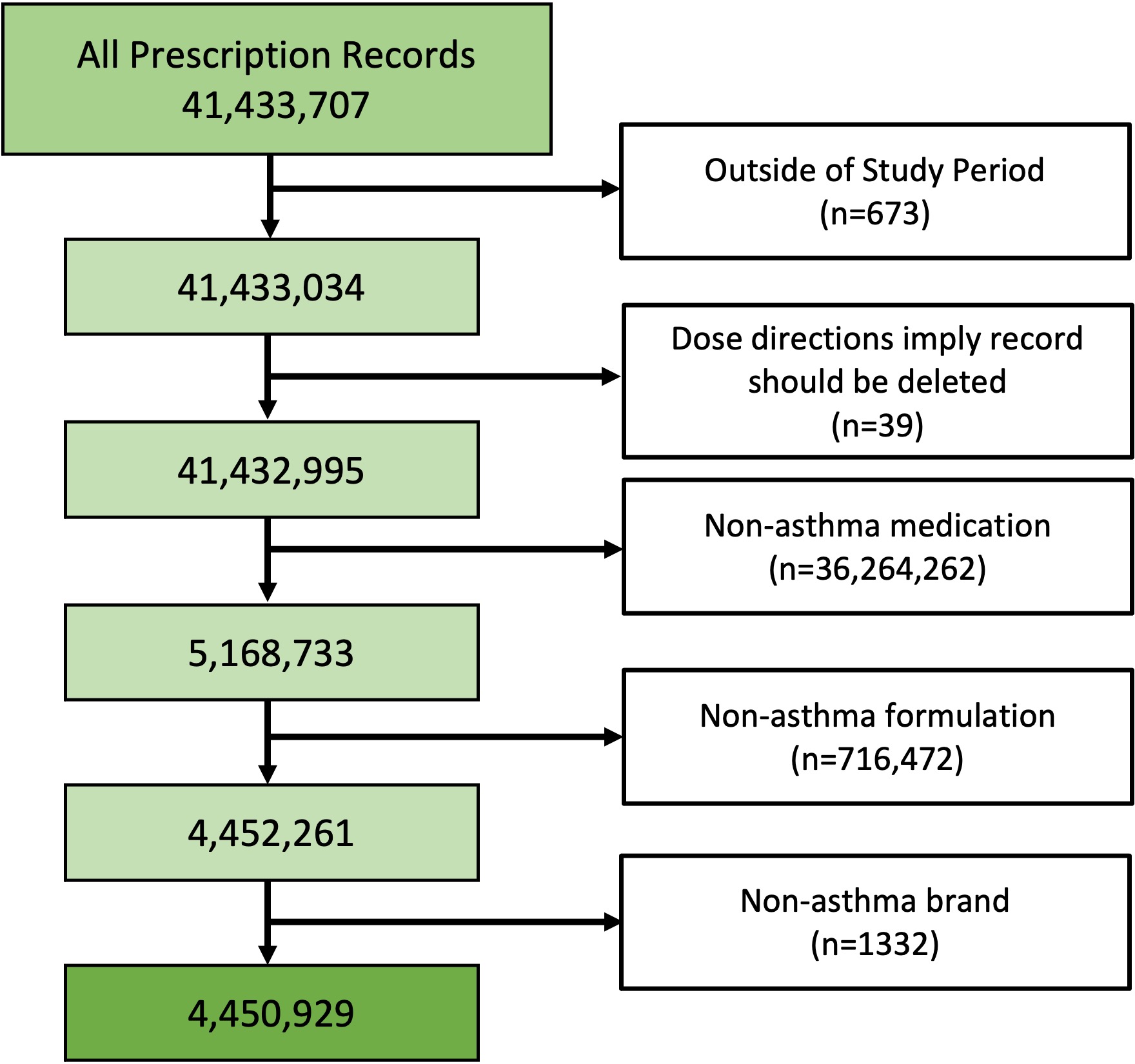

Supplement: Supplementary file 1 — Supplementary Material 1 [file 12890_2022_2189_MOESM1_ESM.jpg]
