# Supplementary material for: Derivation of asthma severity from electronic prescription records using British thoracic society treatment steps
Source: BMC Pulm Med. 2022 Nov 3;22:397. doi: 10.1186/s12890-022-02189-3 (PMC9635147; doi:10.1186/s12890-022-02189-3)
Supplement: Supplementary file 2 — Supplementary Material 2 [file 12890_2022_2189_MOESM2_ESM.docx]

## Appendix A: British Thoracic Society and Scottish Intercollegiate Guidelines Network (BTS/SIGN) 2019 asthma treatment recommendations

| **Step** | **Treatment Options** | **BTS/SIGN Guidelines** (1) **Quote** |
| --- | --- | --- |
| 1 | Low-Strength ICS | *“﻿A reasonable starting dose of inhaled corticosteroids will usually be low dose for adults”* |
| 2 | Low-Strength ICS + LABA | *“﻿The first choice as add-on therapy to inhaled corticosteroids in adults is an inhaled long-acting β2 agonist, which should be considered before increasing the dose of inhaled corticosteroid”* |
| 3 | Medium-Strength ICS,  Medium-Strength ICS + LABA,  Low-Strength ICS + LTRA | *“﻿If there is an improvement when LABA is added, but control remains suboptimal, continue with the LABA and increase the dose of ICS to medium.”*  *“If there is no improvement when a LABA is added, consider stopping the LABA before increasing the dose of ICS”*  *“If there is no improvement following addition of a LABA, consider stopping the LABA and initiating a trial of LTRA”* |
| 4 | Medium-Strength ICS + LTRA,  Medium-Strength ICS + LTRA + add-on therapy,  Medium-Strength ICS + LTRA + LABA,  Medium-Strength ICS + LTRA + LABA + add-on therapy,  High-Strength ICS,  High-Strength ICS + LTRA,  High-Strength ICS + LTRA +add-on therapy,  High-Strength ICS + LABA,  High-Strength ICS + LABA +add-on therapy,  High-Strength ICS + LTRA + LABA,  High-Strength ICS + LTRA + LABA + add-on therapy | *﻿﻿“If asthma control remains inadequate on medium-dose […] of inhaled corticosteroid plus a long-acting β2 agonist or a leukotriene receptor antagonist, the following interventions can be considered:*   - *Increase the inhaled corticosteroids to high dose […]* - *Add a leukotriene receptor antagonist (if not already trialled)* - *Add tiotropium* - *Add a theophylline”* |

Note: ICS = Inhaled CorticoSteroids, LABA = Long-Acting Beta-2 Agonist, LTRA = Leukotriene Receptor Antagonist, BTS = British Thoracic Society, SIGN = Scottish Intercollegiate Guidelines Network. ‘Add-on’ therapies LAMA, theophylline, monoclonal antibody therapy, and maintenance OCS. Guidelines in this table refer to adults; guidance related to paediatric treatment steps has been omitted.

## Appendix B

**UK Asthma Medication Brand and Generic Names, Formulations, and Dosages**

Note: This table is an update of the categorisation used previously by Mukherjee *et al.* (40). The updates include the addition of new brands added to the British National Formulary (brand highlighted in bold) and new therapies approved by NICE (also highlighted in bold, with NICE technical appraisal and evidence summary identifiers included). The formulations and dosages approved for asthma treatment in adults were extracted from the British National Formulary on April 10^th^, 2020, or sourced from (the most recent) previous versions for medications which are no longer recommended.

| **Drug Type** | **Ingredients** | **Brand Names** | **Formulation** | **Medication Strength** |
| --- | --- | --- | --- | --- |
| Short-Acting Beta-2 Agonist (SABA) | Salbutamol / Albuterol | *Generic* | Tablet | 2mg, 4mg |
|  |  |  | Oral Solution | 2mg/5ml |
|  |  |  | Pressurised Inhaler | 100mcg |
|  |  |  | Inhalation Powder | 100mcg, 200mcg |
|  |  |  | Nebulising Solution | 2.5mg/2.5ml, 5mg/2.5ml |
|  |  | Salamol | Pressurised Inhaler | 100mcg |
|  |  |  | Nebulising Solution | 5mg/2.5ml |
|  |  | Ventolin | Infusion Ampoules | 5mg/5ml |
|  |  |  | Injection | 500mcg/1ml |
|  |  |  | Oral Solution | 2mg/5ml |
|  |  |  | Pressurised Inhaler | 100mcg |
|  |  |  | Inhalation Powder | 200mcg |
|  |  |  | Nebules | 2.5mg, 5mg |
|  |  | Ventolin | Nebulising Solution | 5mg/1ml |
|  |  | Airomir | Pressurised Inhaler | 100mcg |
|  |  | Salbulin | Inhalation Powder | 100mcg |
| **Drug Type** | **Ingredients** | **Brand Names** | **Formulation** | **Medication Strength** |
| Short-Acting Beta-2 Agonist (SABA) | Salbutamol / Albuterol | AirSalb | Pressurised Inhaler | 100mcg |
|  |  | Ventmax | Capsule | 4mg, 8mg |
|  |  | Ventolin | Nebulising Solution | 5mg/1ml |
|  |  | Asmasal | Inhalation Powder | 95mcg |
|  |  | Pulvinal Salbutamol | Inhalation Powder | 200mcg |
| Long-Acting Beta-2 Agonist (LABA) | Bambuterol | Bambec | Tablet | 10mg |
|  | Formoterol | *Generic* | Inhalation Powder | 12mcg |
|  |  | Atimos | Pressurised Inhaler | 12mcg |
|  |  | Foradil | Inhalation Powder | 12mcg |
|  |  | Oxis | Inhalation Powder | 6mcg, 12mcg |
|  | Salmeterol | Neovent | Pressurised Inhaler | 25mcg |
|  |  | Serevent | Pressurised Inhaler | 25mcg |
|  |  |  | Inhalation Powder | 50mcg |
|  | Terbutaline | Bricanyl | Tablet | 5mg |
|  |  |  | Injection | 2.5mg/5ml, 500mcg/1ml |
|  |  |  | Inhalation Powder | 500mcg |
|  |  |  | Nebulising Solution | 5mg/2ml |
|  | **Tiotropium [ESNM55**] | **Spiriva Respimat** | Pressurised Inhaler | 2.5mg |
| Long-Acting Muscarinic Antagonists (LAMA) | Ipratropium | *Generic* | Nebulising Solution | 250mcg/1ml, 500mcg/2ml |
|  |  | Atrovent | Pressurised Inhaler | 20mcg |
|  |  | Atrovent | Nebulising Solution | 250mcg/1ml, 500mcg/2ml |
|  |  | **Inhalvent** | Pressurised Inhaler | 20mcg |
|  |  | **Ipravent** | Pressurised Inhaler | 20mcg |

Notes: Highlighted in bold are the brands added to the British National Formulary and therapies approved by NICE (with NICE technical appraisal and evidence summary identifiers included) since the Mukherjee *et al.* (40) study.

| **Drug Type** | **Ingredients** | **Brand Names** | **Formulation** | **Medication Strength** |
| --- | --- | --- | --- | --- |
| Long-Acting Muscarinic Antagonists (LAMA) | Ipratropium | Respontin | Nebulising Solution | 250mcg/1ml, 500mcg/2ml |
|  |  | Atrovent | Nebulising Solution | 250mcg/1ml, 500mcg/2ml |
| LAMA + LABA | Ipratropium + Salbutamol | Ipramol | Nebulising Solution | (200mcg + 1mg) / 1ml |
|  |  | Combivent | Nebulising Solution | (200mcg + 1mg) / 1ml |
| Theophylline | Theophylline | Uniphyllin | Tablet | 200mcg, 300mcg, 400mcg |
|  |  | Nuelin | Tablet | 175 mg, 250 mg |
|  |  | Slo-Phyllin | Tablet | 60mg, 125mg, 250mg |
|  | Aminophylline | Phyllocontin | Tablet | 225mg, 350mg |
|  |  |  | Injection | 250mg/10ml |
| Inhaled Corticosteroid (ICS) | Beclometasone / Beclomethasone | *Generic* | Inhalation Powder | 200mcg |
|  |  | Clenil | Pressurised Inhaler | 50mcg, 100mcg, 200mcg, 250mcg |
|  |  | Qvar | Pressurised Inhaler | 50mcg, 100mcg |
|  |  | Becodisks | Inhalation Powder | 100mcg, 200mcg, 400mcg |
|  |  | Pulvinal Beclometasone | Inhalation Powder | 100mcg, 200mcg, 400mcg |
|  |  | Asmabec | Pressurised Inhaler | 100mcg, 250mcg |
|  |  | Beclazone | Pressurised Inhaler | 50mcg, 100mcg, 200mcg, 250mcg |
|  |  | Filair | Pressurised Inhaler | 50mcg, 100mcg, 250mcg |
|  |  | Aerobec | Pressurised Inhaler | 50mcg, 100mcg, 250mcg |
|  | Budesonide | *Generic* | Nebulising Solution | 250mcg/2ml, 500mcg/2ml, 1mg/2ml |

Notes: Dosage for LAMA+LABA combination treatments are listed LAMA first, LABA second.

Highlighted in bold are the brands added to the British National Formulary since the Mukherjee *et al.* (40) study.

| **Drug Type** | **Ingredients** | **Brand Names** | **Formulation** | **Medication Strength** |
| --- | --- | --- | --- | --- |
| Inhaled Corticosteroid (ICS) | Budesonide | Budelin | Inhalation Powder | 100mcg, 200mcg, 400mcg |
|  |  |  | Inhalation Powder | 200mcg |
|  |  | Pulmicort | Inhalation Powder | 100mcg, 200mcg, 400mcg |
|  |  |  | Respules | 0.5mg, 1mg |
|  | Fluticasone | Flixotide | Pressurised Inhaler | 50mcg, 125mcg, 250mcg |
|  |  |  | Inhalation Powder | 50mcg, 100mcg, 250mcg, 500mcg |
|  |  |  | Nebules | 0.5mg/2ml, 2mg/2ml |
|  | Mometasone | Asmanex | Inhalation Powder | 200mcg, 400mcg |
|  | Ciclesonide | Alvesco | Pressurised Inhaler | 80mcg, 160mcg |
| ICS + LABA | Beclometasone + Formoterol | Fostair | Pressurised Inhaler | 100mcg+6mcg, 200mcg+6mcg |
|  |  |  | Inhalation Powder | 100mcg+6mcg,  200mcg+6mcg |
|  | Budesonide + Formoterol | Symbicort | Pressurised Inhaler | 200mcg+6mcg |
|  |  |  | Inhalation Powder | 100mcg+6mcg, 200mcg+6mcg, 400mcg+12mcg |
|  |  | **DuoResp Spiromax** | Inhalation Powder | 160mcg+4.5mcg, 320mcg+12mcg |
|  | Fluticasone + Formoterol | Flutiform | Pressurised Inhaler | 50mcg+5mcg, 25mcg+5mcg, 250mcg+10mcg |

Notes: Dosage for ICS+LABA combination treatments are listed ICS first, LABA second.

Highlighted in bold are the brands added to the British National Formulary since the Mukherjee *et al.* (40) study.

| **Drug Type** | **Ingredients** | **Brand Names** | **Formulation** | **Medication Strength** |
| --- | --- | --- | --- | --- |
| ICS + LABA | Fluticasone + Salmeterol | Seretide | Pressurised Inhaler | 50mcg+25mcg, 125mcg+25mcg, 250mcg+25mcg |
|  |  | Seretide | Inhalation Powder | 100mcg+50mcg,  250mcg+50mcg, 500mcg+50mcg |
|  |  | Airflusal | Pressurised Inhaler | 125mcg+25mcg, 250mcg+25mcg |
|  |  |  | Inhalation Powder | 500mcg+50mcg |
|  |  | **Sirdupla** | Pressurised Inhaler | 250mcg+25mcg |
|  | Fluticasone + Vilanterol | Relvar Ellipta | Inhalation Powder | 92mcg+22mcg, 184mcg+22mcg |
| Leukotriene Receptor Antagonist (LTRA)* | Montelukast | *Generic* | Tablet | 4mg, 5mg, 10mg |
|  |  |  | Sachet for Solution | 4mg |
|  |  | Singulair | Tablet | 5mg, 10mg |
|  |  |  | Sachet for Solution | 4mg |
|  | Zafirlukast | *Generic* | Tablet | 10mg, 20mg |
|  |  | Accolate | Tablet | 10mg, 20mg |
|  | Cromolyn / Sodium Cromoglicate | Intal | Pressurised Inhaler | 5mg |
|  | Nedocromil | Tilade | Pressurised Inhaler | 2mg |

Notes: Dosage for ICS+LABA combination treatments are listed ICS first, LABA second.

Mast Cell Stabilisers Cromolyn and Nedocromil have been included under LTRAs, although they have a distinct mode of action

## Highlighted in bold are the brands added to the British National Formulary since the Mukherjee *et al.* (40) study.

## Appendix C

### **Keywords for** **Identifying Asthma Medications**

Box A: Corticosteroid Asthma Therapy Exclusion Formulation, Delivery, and Indication Terms

| "NASAL", "NOSE", "NOSTRIL", "NASULE", "HAYFEVER", "EYE", "EAR", "DROP", "TONGUE", "FOAM", "ENEMA", "RECTAL", "SUPPOSITOR", "CREAM", "OINTMENT", "ULCER", "SKIN", "PATCH", "APPLY" |
| --- |

Box B: Corticosteroid, Long-Acting Beta-2 Agonist, Long-Acting Muscarinic Antagonist, and Leukotriene Receptor Antagonist Therapy Brands to be Excluded due to Non-Asthma Indication

| "NASONEX", "FLIXONASE", "ANORO ELLIPTA", "SUMATRIPTAN", "AVAMYS", “RHINOCORT", "NASOBEC", "NASOFAN", "RYNACROM", "PIRINASE", "SPIOLTO", "DYMISTA" , "POLLENASE", "VIVIDRIN", "DUAKLIR", "SEEBRI", "ULTIBRO", "PRED FORTE", "TRELEGY", "TRIMBOW", "BRALTUS", "RINATEC", "ENTOCORT", "BENACORT", "AIRCORT", "BUDEFLAM", "BUDENOFALK", "CORTIMENT", "JORVEZA", "AZELASTINE", "CUTIVATE", "ELOCON", "NALCROM", "CATACROM", "ASPIRE", "OPTICROM", "OPTREX", "BECONASE", "MURINE", "ACLIDINIUM", "GENUAIR", "OLADATEROL", "YANIMO" |
| --- |

## Appendix D

**Daily Medication Dose Frequency Keywords and Observed Incidence**

| **Daily Dose Frequency** | **Key Words** | | |
| --- | --- | --- | --- |
| Once | "ONCE","O-D", "O.D" | | |
|  | "DAILY", "EVERY DAY", "EACH DAY" | WITHOUT | "TWICE", "TWO TIMES", "2 TIMES", "TD", "TID", "BID", "BD", "B-D", "B.D", “FOUR TIMES”, “4 TIMES”, “QID” |
|  | “MORN” |  | “NIGHT”, “EVE”, “BEDTIME” |
|  | “MANE” |  | “NOCTE” |
|  | ‘NOCTE” |  | “MANE” |
|  | “AM” |  | “PM” |
|  | “PM” |  | “AM” |
|  | “A.M” |  | “P.M” |
|  | “P.M” |  | “A.M” |
| Twice | "TWICE", "TWO TIMES", "2 TIMES", "TD", "TID", "BID", "BD", "B-D", "B.D" | | |
|  | “MORN” | WITH | “NIGHT”, “EVE”, “BEDTIME” |
|  | “AM” |  | “PM” |
|  | “A.M” |  | “P.M” |
|  | “MANE” |  | “NOCTE” |
| Four Times | "QID", "FOUR TIMES", "4 TIMES" | | |
| Unknown | N/A | | |

## Appendix E

Dosage (micrograms per day) categories for Corticosteroid and combination Long-Acting Beta-2 Agonist inhalers

| **ICS Drug** | **Brand Names** | **Low Daily ICS Dose** | **Medium Daily ICS Dose** | **High Daily ICS Dose** |
| --- | --- | --- | --- | --- |
| Beclometasone | Clenil, Becodisks, AeroBec, Beclazone, Filair * | 1-400mcg | 401-800mcg | 801-3200mcg |
|  | Qvar, Pulvinal Beclometasone | 1-200mcg | 201-400mcg | 401-1600mcg |
|  | Asmabec | 1-200mg | 201-400mg | N/A |
| Budesonide | Budelin | N/A | 400-800mcg | 801-3200mcg |
|  | Pulmicort * | 1-400mcg | 401-800mcg | 801-3200mcg |
| Fluticasone | Flixotide* | 1-200mcg | 201-500mcg | 501-2000mcg |
| Mometasone | Asmanex Twisthaler * | 1-400mcg | 401-800mcg | N/A |
| Ciclesonide | Alvesco * | 1-160mcg | 161-320mcg | N/A |
| Beclometasone + Formoterol | Fostair * | 1-200mcg | 201-400mcg | 401-1600mcg |
| Budesonide + Formoterol | Symbicort | 1-400mcg | 401-800mcg | 801-3200mcg |
|  | DuoResp Spiromax * | 1-320mcg | 321-640mcg | 641-2560mcg |
| Fluticasone + Formoterol | Flutiform * | 1-200mcg | 201-500mcg | 501-2000mcg |
| Fluticasone + Salmeterol | Seretide * | 1-200mcg | 201-500mcg | 501-2000mcg |
|  | Sirdupla | N/A | 250-500mcg | 501-2000mcg |
|  | AirFluSal Forspiro | N/A | N/A | 500-2000mcg |
| Fluticasone + Vilanterol | Relvar Ellipta * | N/A | 46-92mcg | 93-368mcg |

Notes:

ICS = Inhaled CorticoSteroid, LABA = Long-Acting Beta-2 Agonist

Boundaries between low, medium, and high-dose ranges are based on reference values listed in the 2019 BTS/SIGN Guidelines (1).Generic medications or records with unlisted brand were assigned to the category of the brand name group highlighted by asterisk.

## Appendix F

**Prescription Record Processing Report**

The prescriptions dataset contained 41,433,707 entries (prescriptions) for 671,304 individuals, with linked dispensing data. 673 records were removed that were dated outside of the study period (January 31^st^, 2009, to March 31^st^, 2017), and an additional 39 records were excluded after linking, however, as the dose directions indicated that the record should be deleted due to an error, leaving 41,432,995 records remaining (671,298 individuals).

5,168,733 prescriptions were recognised as potential asthma medications (ICS, Combination ICS+LABA, LABA, LAMA, LTRA, SABA, and theophylline), according to identified brand names and active ingredients. Records with formulation listed as a spray (n=689,085) or a drop (n=22,938) were excluded, as well as a further 4449 records containing one or more formulation exclusion keywords in the dose directions, leaving 4,452,261 records (exclusion flowchart illustrated below). 1332 records were excluded which were identified as branded medications used to treat indications other than asthma (and had not been previously excluded). 4,450,929 prescription records remained for 160,178 unique individuals.

[Insert Appendix F Figure here]

2637 ICS records were recategorised as non-inhaled medications, leaving 1,627,846 ICS and combination ICS+LABA prescriptions. The modal dose frequency by medication (ingredients) was imputed when a value could not be extracted: once daily for Ciclesonide and Fluticasone Vilanterol (n=1210), else twice daily (n=162,115). The modal dose quantity by medication was also imputed when a value could not be extracted: one dose at each daily dose time for Budesonide, Ciclesonide, Fluticasone Vilanterol, Fluticasone Salmeterol, and Mometasone (n=71,880), else two (n=103,634).

| **(N=1,627,846)** | **Percentage of Prescriptions Before Imputation** | **Percentage of Prescriptions After Imputation** |
| --- | --- | --- |
| **Dose Frequency** | | |
| Once | 2.3 | 2.4 |
| Twice | 87.4 | 97.4 |
| Four Times | 0.2 | 0.2 |
| Unknown | 10.0 | - |
| **Dose Quantity** | | |
| One | 35.8 | 40.2 |
| Two | 53.0 | 59.3 |
| Three | 0.2 | 0.2 |
| Four | 0.3 | 0.3 |
| Unknown | 10.8 | - |

220 prescriptions had extracted strength values which were not listed on the lookup table presented in Appendix A and were thus excluded. This left 1,627,626 prescriptions for 91,334 unique individuals. No strength value could be extracted for 40.2% of prescriptions. As such, we added to the extraction that the microgram values previously specified could also be followed by “CLICKHALER”, “ACCUHALER”, “EVOHALER”, or “TURBOHALER”, or preceded by “QVAR”, “SERETIDE”, “SERETIDE MDI”, “INHAL”, or “ALVESCO”. After this, only 8.4% remained missing. (n=136,164), and the modal value by medication was imputed.
